# *In Silico* Approach for Prediction of Antifungal Peptides

**DOI:** 10.3389/fmicb.2018.00323

**Published:** 2018-02-26

**Authors:** Piyush Agrawal, Sherry Bhalla, Kumardeep Chaudhary, Rajesh Kumar, Meenu Sharma, Gajendra P. S. Raghava

**Affiliations:** ^1^Council of Scientific and Industrial Research, Institute of Microbial Technology, Chandigarh, India; ^2^Center for Computational Biology, Indraprastha Institute of Information Technology, New Delhi, India

**Keywords:** antimicrobial peptides, antifungal peptides, amino acid composition, support vector machine, motifs

## Abstract

This paper describes *in silico* models developed using a wide range of peptide features for predicting antifungal peptides (AFPs). Our analyses indicate that certain types of residue (e.g., C, G, H, K, R, Y) are more abundant in AFPs. The positional residue preference analysis reveals the prominence of the particular type of residues (e.g., R, V, K) at N-terminus and a certain type of residues (e.g., C, H) at C-terminus. In this study, models have been developed for predicting AFPs using a wide range of peptide features (like residue composition, binary profile, terminal residues). The support vector machine based model developed using compositional features of peptides achieved maximum accuracy of 88.78% on the training dataset and 83.33% on independent or validation dataset. Our model developed using binary patterns of terminal residues of peptides achieved maximum accuracy of 84.88% on training and 84.64% on validation dataset. We benchmark models developed in this study and existing methods on a dataset containing compositionally similar antifungal and non-AFPs. It was observed that binary based model developed in this study preforms better than any model/method. In order to facilitate scientific community, we developed a mobile app, standalone and a user-friendly web server ‘Antifp’ (http://webs.iiitd.edu.in/raghava/antifp).

## Introduction

Despite tremendous advances in the field of antibiotics; the morbidity and mortality are quite high due to invasive fungal infections (Kanafani and Perfect, 2008). The major fungal species like *Candida, Aspergillus, Pneumocystis*, and *Cryptococcus* spp. (Sanglard, 2016) are responsible for causing 1.4 million deaths worldwide per year (Brown et al., 2012). Drug or antibiotic resistance is one of the major causes of millions death per year due to antifungal infections (Haegerstrand et al., 1992; Miceli et al., 2011). In order to overcome the problem of drug resistance, researchers are exploring alternatives to antibiotics (small molecules). One of the alternates to small chemical-based drugs is peptide-based therapeutics. It is safer and more effective than traditional therapeutics and provides effective arms to researchers fight against fungus. One can understand importance of peptide-based therapeutics from the fact that in the last one decade, number of peptide resources has been developed (Kapoor et al., 2012; Novković, 2012; Gautam et al., 2014; Waghu et al., 2014; Kumar et al., 2015; Agrawal et al., 2016; Mathur et al., 2016; Singh et al., 2016; Wang et al., 2016; Usmani et al., 2017).

One of the major classes of peptide-based therapeutics comes from the antimicrobial peptides (AMPs). AMPs can be classified into different kinds of peptides like antibacterial, antiviral, antifungal, antiparasitic, etc. In past, extensive efforts have been made to the study and development of novel AMPs. More than 2300 AMPs are reported in the different AMP databases. It includes more than 100 peptide-based drugs that are present in the market and approximately 600 are in pre-clinical stage (Craik et al., 2013). Though AMPs can be used to treat fungal infection but lack of specificity reduces their potential. Thus, there is a strong need to design antifungal peptides (AFPs) to treat fungal infections as existing drugs (e.g., Amphotericin B deoxycholate, Voriconazole, Fluconazole, Itraconazole, Terbinafine, Posaconazole) fail due to drug resistance. AFPs have the ability to kill fungus as it disrupts membrane physiology of fungus (Eckert, 2011; Fjell et al., 2011; Wimley and Hristova, 2011). AFPs have been found to be very effective in several cases, for example, in the case of azole-resistant *Candida* species, Brevinin-1BYa is a highly effective α-helical peptide (Zubkov et al., 2000). P113 is one of the histidine variants, which has shown efficacy against *C. albicans in vitro* (Oppenheim et al., 1988).

Numerous methods have been developed in past for predicting and designing AMPs (antibacterial, antiviral, etc.) such as template-based method (Pag et al., 2008; Robinson, 2011), docking simulations (Schneider and Fechner, 2005; Jorgensen, 2009), hidden Markov model (HMM) (Fjell et al., 2007, 2008) and sequence-based methods (Lata et al., 2007, 2010). Recently, existing methods developed for predicting/designing antimicrobial have been evaluated and reviewed in depth (Fjell et al., 2011; Porto et al., 2017a). In comparison to AMPs, limited attempts have been made to understand and develop methods for predicting AFPs. ClassAMP is one of the methods which predicts the given peptide as Antibacterial, Antiviral, Antifungal, etc. with a probability score (Joseph et al., 2012). Another such method is iAMP-2L which first predicts the antimicrobial activity and then classifies them into specific antimicrobial class (Xiao et al., 2013). In the current study, an attempt has been made to develop models using machine learning techniques for discriminating AFPs from natural peptides and other AMPs (Mousavizadegan and Mohabatkar, 2016). The machine learning techniques derive rules from experimentally validated antifungal and non-AFPs to discriminate two classes of peptides. These rules are used to predict antifungal properties of a peptide. One of the challenges for designing any prediction method is the compilation of negative dataset (Saha and Raghava, 2006; Chou, 2011; Porto et al., 2017c). Therefore, in our study for designing negative dataset, we selected those AMPs as negative peptides, which do not show any antifungal activity, since there is no dedicated database that maintains non-AFPs. In addition, we also generated random peptides from proteins in SwissProt database and used them as non-AFPs. The overall objective of this study was to develop an *in silico* prediction method which can discriminate AFPs from non-AFPs with high accuracy, similar to Chou’s five-step rule (Chou, 2011). We also developed a mobile app and standalone software to facilitate users in predicting AFPs.

## Materials and Methods

### Datasets Preparation

We extracted 1585 exclusive AFPs from the AMP-maintaining database DRAMP (Fan et al., 2016). Peptides containing non-natural amino acids (BJOUZX) and repeated sequences were removed which led to the 1459 unique AFPs. We created three datasets, first is our main dataset termed as “Antifp_Main” and two alternate datasets termed as “Antifp_DS1” and “Antifp_DS2.” During dataset creation, the range of peptide length was kept same in both positive and negative datasets. We generated different bins (e.g., 0-10, 11-20, 20-30, etc. till 90-100) and ensured that same number of peptides are present in the bin of both datasets. Details about three different datasets used in the study are described below.

**(i) Antifp_Main**
  Our main dataset consists of 1459 positive peptides, which are exclusive AFPs, and 1459 negative peptides, which were generated mixing peptides possessing antimicrobial function other than antifungal and peptides generated randomly from SwissProt.
**(ii) Antifp_DS1**
  This first alternate dataset consists of 1459 exclusive AFPs as the positive dataset and an equal number of peptides, which possess antimicrobial activity other than antifungal (e.g., antibacterial, antiviral, etc.) as the negative dataset.
**(iii) Antifp_DS2**
  We also developed a second alternate dataset in which exclusive AFPs were taken as positive peptides and keeping in mind the similar length distribution, an equal number of negative peptide sequences was generated randomly from SwissProt. This kind of approach has been used earlier (Chaudhary et al., 2016).

### Internal and External Validation

The datasets were randomly divided into two parts (i) training dataset, which comprises of 80% data (1168 positive and negative sequences) and (ii) validation dataset with 20% data (291 positive and negative sequences). In case of internal validation, we developed and evaluate prediction models using fivefold cross-validation techniques. Here, sequences present in the dataset are divided randomly into five different sets, out of which any four sets out of five are used for training and the remaining fifth set is used for testing. In the process, each set is used once for testing by repeating the process five times, and the final result is calculated by averaging the performance of all the five sets. The validation of any prediction method plays a very significant role in its evaluation. We evaluated the performance of all the models on validation dataset, termed as external validation.

### Dataset for Additional Benchmarking

Previous studies have shown that discriminating between the peptides with the same composition but different activity is a big challenge (Loose et al., 2006; Porto et al., 2017b). In order to evaluate the performance of models developed in this study and methods developed in the past, we create a dataset Antifp_hard that contain compositionally similar antifungal and non-anti-fungal peptides. The positive set of Antifp_hard dataset contains exclusive AFPs used in the validation set. The negative set or non-AFPs in Antifp_hard is obtained from AMPs, which have highest compositional similarity with exclusive AFPs. In order to identify compositionally similar peptides, we compute Euclidean distance between composition of two peptides (Kumar et al., 2008) and identify peptides having minimum Euclidean distance.

### Positional Residues Preference in AFPs

We calculate positional preferences of all types of residues at different positions in both the terminus (N and C) in the form of quantitative matrices (QMs). This kind of approach has been used in previous studies also for computing positional preferences (Gupta et al., 2013). It shows the propensity of each amino acid at each position in both the dataset, positive as well as negative. We generated the QM for first 15 residues from N-terminal and first 15 residues from the C-terminal generating matrix of dimension 20 × 15.

### MERCI Motifs Analysis

We also looked for various common patterns or motifs present in AFPs and non-AFPs and to identify those patterns/motifs; we have used MERCI program (Vens et al., 2011). The default criteria were set while running the program. The motifs were extracted from all the datasets, i.e., Antifp_Main, Antifp_DS1, and Antifp_DS2. This program compares both the positive and negative peptides for extracting motifs. In order to know motifs, present in AFPs and non-AFPs, we used two-step strategy. In this method, we first provided AFPs as positive input and non-AFPs as a negative input. In the next step, we reversed the order of input where non-AFPs were given as positive input and AFPs were given as negative input. The same procedure was followed for all the datasets. Finally, we obtained several numbers of motifs present in AFPs and non-AFPs, which can be utilized further to scan peptides for the presence of AFP-specific motif.

### Input Features for Prediction

**(i) Amino acid composition-based model**
In earlier studies, people have shown that amino acid composition can be used for classifying various peptides and for developing prediction methods using machine-learning techniques (Raghava and Han, 2005). The amino acid composition tells us about the fraction of each amino acid type within a peptide. The vector of dimension 20 was obtained when the amino acid composition for both AFPs and non-AFPs was calculated by using the following equation:

(1) $$\begin{array}{c} \mathrm{Composition}(i)=\frac{R_{i}}{N}*100 \end{array}$$

Here, Composition (i) is the percent composition of amino acid (i); R_i_ is the number of residues of type i, and N represents the total number of peptide’s residues.
**(ii) Dipeptide composition-based model**
The dipeptide composition provides the composition of the residues present in a pair (e.g., A-A, A-L, etc.) in the peptide, and used to convert the variable length of peptides to fixed length feature vector size of 400. It summarizes information about the amino acid’s fraction as well as their local order. Dipeptide composition is calculated using following equation:

(2) $$\begin{array}{c} Dipeptide\;fraction(i)=\;\frac{TotalnumberofDipeptide(i)}{Totalnumberofallpossibledipeptides}*100 \end{array}$$

Where dipeptide (i) is 1 out of 400 dipeptides.
**(iii) Split composition-based model**
We also compute amino acid and dipeptide composition of N-terminus and C-terminus residues; first 5, 10, and 15 residues from N-terminus and the last 5, 10, and 15 residues from the C-terminus. Also, we joined the terminal residues like N5C5, N10C10, and N15C15 and checked the performance of combination.
**(iv) Binary profile based model**
In this study, length of antifungal and non-AFP is variable, thus it is difficult to generate fixed length pattern. Thus we extract fixed length segment from either N-terminus or C-terminus of the peptide to generate fixed length binary profile (Lata et al., 2010). A vector of dimension 20 represented each amino acid in segment obtained from terminal residues. We generated binary profiles for first 5, 10, and 15 N-terminus residues and for the last 5, 10, and 15 residues from the peptide C-terminus. We also created the binary profile for the N5C5, N10C10, and N15C15 residues of peptides by combining N- and C-terminus residues. The binary profile has been used heavily in a number of studies for predicting functional properties of peptides (Xiao et al., 2009; Gautam et al., 2013; Chaudhary et al., 2016).
**(v) Calculation of Mass, Charge, and pI value of peptide**
Mass, charge, and pI value of peptide were calculated using R package “*peptides*” (Osorio et al., 2015), which is specifically designed for the quick and easy calculation of different AMPs features. This would help in their better classification and design. Default parameters were used for calculation of mass, charge, and pI values and the values were used as features along with the amino acid composition on which the best performance was found. We wanted to check whether adding these properties would help in further increasing the performance of a model. Thus, the dimension of our composition based model increased from 20 to 23 to adjust above three properties.

### Machine Learning Approaches

We used different machine learning techniques for developing prediction models. The approaches are as follows:

**Support Vector Machine**
We used SVM*^light^* Version 6.02 package of SVM for building the prediction models, which is a highly successful machine learning classifier (Schölkopf et al., 1999). This package consists of various kernels and machine learning was performed using these kernels where each input dot is transformed into non-linear kernel function. SVM’s RBF kernel was used here at various parameters; g € [10^-4^**–**10], c € [1–15], j € [1–5] for optimizing the SVM performance to obtain the best performances. RBF kernel is squared exponential kernel which provides more functional space and is more flexible than a linear or polynomial kernel and hence provides much better results. Sets of input features with a fixed length are required for training, thus imposing a strategy for encapsulating the overall information about proteins/peptides of fixed length format. Different features like the binary profile, different composition methods (amino-acid, dipeptide) are used to get fixed length format from protein/peptide sequences of variable length. After training, learned models could be employed for the prediction of unknown examples.
**WEKA Classifiers**
  We used different classifiers incorporated in WEKA suite for building training models, which could be used for the prediction of unidentified examples. The different classifiers we used include Random Forest, J48, SMO, and Naïve Bayes (Rudensky et al., 1991). Parameters were tuned while performing the different machine learning techniques and the results obtained on best parameters were reported.

### Performance Measure

Broadly, the performance of any classification is measured using two type measures call threshold-dependent and threshold-independent. In this study, we used both types of measure to evaluate the performance of models. In case of threshold-dependent parameters, we compute performance of a model in following terms; Sensitivity (Sen), Specificity (Spc), Accuracy (Acc), and Matthews correlation coefficient (MCC). Following equations are commonly used for computing, threshold-dependent measures.

(3) $$\begin{array}{c} Sensitivity=\frac{TP}{PS}\times 100 \end{array}$$

(4) $$\begin{array}{c} Specificity=\frac{TN}{NS}\times 100 \end{array}$$

(5) $$\begin{array}{c} Accuracy=\frac{TP+TN}{PS+NS}\times 100 \end{array}$$

(6) $$\begin{array}{c} MCC=\frac{1-(\frac{FN}{PS}+\frac{FP}{NS})}{\sqrt{\left(1+\frac{FP-FN}{PS})\times (1+\frac{FN-FP}{NS}\right)}} \end{array}$$

Where *TP* represents correctly predicted positive, *TN* represents the negative examples, *PS* represents total sequences in positive set, *NS* represents total sequences in negative set, *FP* represents actual negative examples which have been wrongly predicted as positive and *FN* represents wrongly predicted positive examples. This is a well-established method of measuring performance and has been used earlier in many studies (Porto et al., 2017b).

### Case Study

Although we validated our model on the independent dataset, we also checked the performance of our model on the recently discovered AFPs.

We obtained peptides from different study performed by Datta et al. (2016), Li et al. (2016), and Garrigues et al. (2017). Therefore, to prevent biases we have taken peptides from different studies and checked the performance of our model on these peptides.

## Results

### Analysis of Residues Composition

**FIGURE 1:**
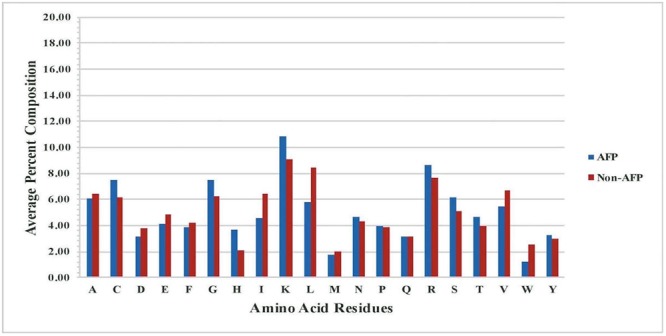
Comparison of percent average amino acid composition of the AFPs- and non- AFPs in Antifp_Main dataset.

It is important to analyze AFPs to understand their nature before we develop *in silico* models for prediction. As all peptides are made of 20 types of residues, it is important to examine the frequency of each type of residues in AFPs. Thus, we computed and compared the amino acid composition of AFPs and non-AFPs of our main dataset, Antifp_Main. The analysis showed that certain residues like C, G, H, K, R, and S, are more abundant or frequent in AFPs whereas non-AFPs are dominated by residues like A, D, E, I, L, V, and W (**Figure 1**). Presence of residues like C, K, R makes AFPs positively charged and cationic in nature. These peptides are divided in two different classes (i) membrane traversing peptides, which forms a pore or act on specific target like chitin synthesis, and (ii) non-membrane traversing peptides, which interacts with the negatively charged fungal membrane and carry out cell lysis (Neelabh et al., 2016).

Likewise, in Antifp_DS1, residues like C, G, H, K, R, S, and T were significantly abundant in AFPs and can be used to discriminate AFPs with other AMPs which are predominant with residues like D, E, I, L, V, and W (Supplementary Figure S1A). In case of Antifp_DS2, we found the abundance of residues like C, H, K, and R whereas non-AFPs contain mainly A, D, E, I, L, and V (Supplementary Figure S1B).

First 15 N and C-terminal residues amino acid composition was also calculated for the three datasets, Antifp_DS1, Antifp_DS2, and Antifp_Main dataset and has been shown in Supplementary Figures S2A, S3A, S4A and for C-terminal residue composition in Supplementary Figures S2B, S3B, and S4B, respectively.

### Positional Residues Preference in AFPs

**FIGURE 2:**
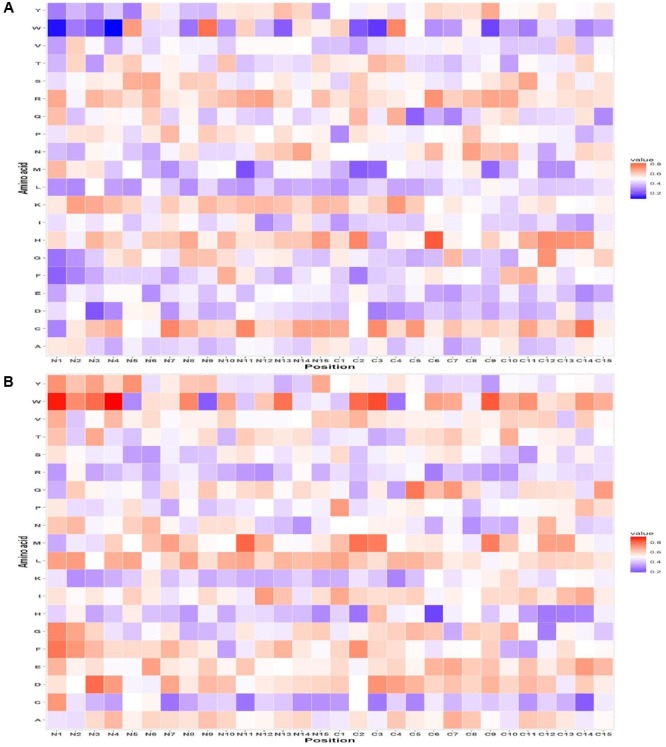
Heat map illustrating the positional preference of each type of residue at (first 15 positions) N and C-terminus **(A)** positive and **(B)** negative data of Antifp_Main dataset.

The probability of residue R is highest at 1^st^ position followed by V and K at the 2^nd^ and 3^rd^ position in AFPs. Similarly, at C-terminus, residue C was highly preferred at the 1^st^ and 3^rd^ position and H at 2^nd^ position in AFPs (**Figure 2A**) as compared to non-AFPs (**Figure 2B**). Likewise, positional residue preference from the QM for Antifp_DS1 and Antifp_DS2 positive and negative data is given in Supplementary Figures S5A,B and S6A,B, respectively.

### Motif Analysis Using MERCI

In order to identify pattern/motifs present in AFPs or non-AFPs, we used software MERCI suite. Sequence analysis of Antifp_Main dataset revealed 13 exclusive motifs for positive and 11 for the negative dataset. Some of the exclusive motifs that are present only in AFPs are “CFCT,” “RCFC,” “NCAS,” “CASV,” etc. whereas motifs present exclusively only in non-AFPs are “CGNTK,” “GNTK,” “NTKH,” etc. (Supplementary Table S1). Similarly, we also extracted exclusive motifs present in positive and negative peptides for Antifp_DS1 (Supplementary Table S2) and Antifp_DS2 (Supplementary Table S3).

### Performance of Various Machine Learning Techniques

Different machine learning approaches like SVM^light^, Random Forest (RF), Naïve Bayes, J48, and SMO were used in the study to generate models on different input features for distinguishing AFPs from non-AFPs. The results are explained in detail in the following sections.

### Amino Acid Composition Based SVM Model

We developed the prediction model using different classifiers like SVM, Random Forest, SMO, Naïve Bayes and J48 on amino acid composition as an input feature. For Antifp_Main, the highest accuracy of 88.27%, MCC of 0.77 and ROC of 0.94 was obtained for training dataset and for validation dataset the accuracy of 86.25%, MCC of 0.73 and ROC of 0.94 was achieved (**Table 1**). In the case of Antifp_DS1, the accuracy of 86.26% with MCC and ROC values 0.73 and 0.93 respectively for the training dataset and accuracy of 85.91%, MCC of 0.72 and ROC of 0.93 for the validation dataset was observed (Supplementary Table S4). For Antifp_DS2 (best dataset obtained after repeating 100 times prediction) we achieved the accuracy of 92.81% and MCC and ROC value 0.86 and 0.97 respectively for the training dataset and accuracy of 90.38%, MCC value 0.81 and ROC of 0.96 for the validation dataset (Supplementary Table S5).

**Table 1:** The performance of different machine learning techniques based models on Antifp_Main dataset developed using amino acid composition of peptides.

	Parameter	Main Dataset				Validation Dataset			
		Sen	Spc	Acc	MCC	ROC	Sen	Spc	Acc	MCC	ROC
SVM	g = 0.01, c = 5, j = 4	88.61	87.93	88.27	0.77	0.94	86.60	85.91	86.25	0.73	0.94
Random Forest	Ntree = 130	87.84	86.64	87.24	0.74	0.93	86.94	80.76	83.85	0.68	0.91
SMO	g = 0.001, c = 2	87.84	82.11	84.97	0.70	0.84	88.32	81.44	84.88	0.70	0.84
J48	c = 0.1, m = 7	80.39	80.65	80.52	0.61	0.82	82.82	81.44	82.13	0.64	0.84
Naïve Bayes	Default	76.46	75.86	76.16	0.52	0.80	74.91	78.01	76.46	0.53	0.81

*Sen, Sensitivity; Spc, Specificity; Acc, Accuracy; MCC, Matthews Correlation Coefficient; ROC, Receiver Operating Characteristic.*

**FIGURE 3:**
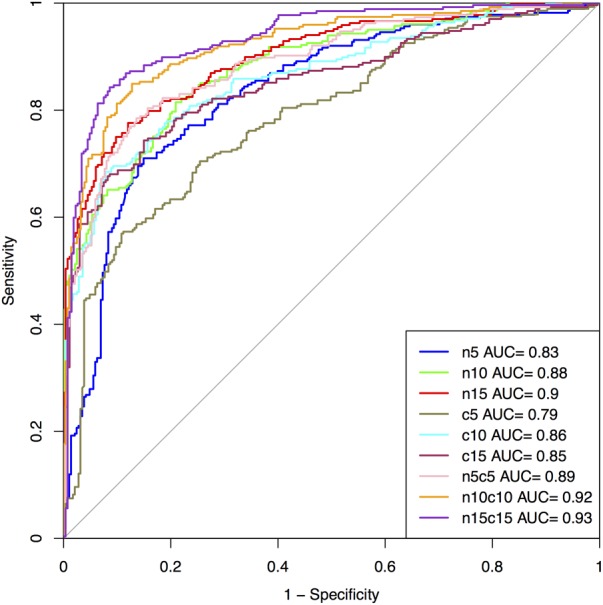
The performance of models on Antifp_Main dataset in term of ROC curves, models were developed using composition features of peptides.

The performance on the first 5, 10, and 15 residues of N and C-terminus as well as their combined form (N5C5, N10C10, and N15C15) of SVM based model is summarized in **Figure 3** and Supplementary Table S6 for Antifp_Main. Similarly, results on different terminus residues obtained by the SVM based model for Antifp_DS1 and Antifp_DS2 has been shown in Supplementary Tables S7 and S8, respectively.

### Dipeptide Composition Based SVM Model

The dipeptide is a comprehensive feature as compared to amino acid composition alone because it encloses the overall information of the amino acids fraction and their local order. This feature has been used in previous studies to discriminate two types of proteins and peptides (Petrilli, 1993) Thus, SVM model was developed based utilizing dipeptide composition as an input feature. In Antifp_Main, maximum accuracy of 86.77%, MCC of 0.74 and ROC of 0.94 was achieved for training dataset and for validation dataset we achieved the accuracy of 88.66%, MCC of 0.77 and ROC of 0.95 (**Table 2**). In Antifp_DS1, we observed the maximum accuracy of 87.20% with MCC and ROC values 0.74 and 0.94 respectively for the training dataset and accuracy of 86.60%, MCC value 0.73 and ROC of 0.94 for the validation dataset (Supplementary Table S9). In the case of Antifp_DS2, we found the maximum accuracy of 91.87%, MCC of 0.84 and ROC of 0.96 for the training dataset and accuracy of 92.10%, MCC value 0.84 and ROC of 0.96 for the validation dataset (Supplementary Table S10).

**Table 2:** The performance of SVM based models on Antifp_Main dataset, where models were developed using dipeptide composition of whole peptide and part of peptides.

	Parameter			Main Dataset					Validation Dataset				
	g	c	j	Sen	Spc	Acc	MCC	ROC	Sen	Spc	Acc	MCC	ROC
DPC	0.005	2	3	88.53	85.02	86.77	0.74	0.94	89.69	87.63	88.66	0.77	0.95
N5	0.001	1	2	77.94	73.88	75.91	0.52	0.85	82.61	75.69	79.08	0.58	0.87
N10	0.0005	8	2	78.74	79.77	79.26	0.59	0.87	86.63	81.21	84.01	0.68	0.90
N15	0.001	3	3	81.97	80.70	81.33	0.63	0.89	85.07	81.58	83.33	0.67	0.90
C5	0.0005	2	3	70.35	75.45	72.89	0.46	0.81	71.17	79.09	75.18	0.50	0.81
C10	0.001	5	1	80.89	74.01	77.43	0.55	0.87	79.71	76.16	77.92	0.56	0.85
C15	0.001	2	2	80.69	76.33	78.51	0.57	0.86	79.93	77.90	78.92	0.58	0.86
N5C5	0.0005	1	2	78.39	80.13	79.29	0.59	0.87	83.02	83.97	83.51	0.67	0.90
N10C10	0.001	1	2	84.65	78.84	81.73	0.64	0.90	87.87	83.33	85.56	0.71	0.92
N15C15	0.001	1	2	84.97	85.09	85.03	0.70	0.92	85.39	86.52	85.96	0.72	0.93

### Binary Profile Based SVM Model

An SVM based model was developed using the binary profile as an input feature. In the case of a binary profile based model developed using N15C15 achieved maximum accuracy of 84.88% with MCC of 0.70 and ROC of 0.92 on training dataset. This model achieved an accuracy of 84.64% with MCC of 0.69 and ROC of 0.92 on validation dataset (**Table 3**). It is important to note that performance of this model is nearly same for training and validation dataset. All these models were developed on Antifp_Main dataset that contains AFPs and negative set of peptides (random and AMPs). We also developed and evaluate models on dataset Antifp_DS1 and achieved maximum accuracy (N15C15) of 84.44% with MCC of 0.69 and ROC of 0.92 for training dataset and accuracy of 81.63% with MCC of 0.63 and ROC of 0.92 on for validation dataset (Supplementary Table S11). In case of Antifp_DS2, we achieved maximum accuracy (N15C15) of 92.32% with MCC of 0.85 and ROC of 0.97 for training dataset and accuracy of 92.70% with MCC of 0.85 and ROC of 0.97 for validation dataset (Supplementary Table S12).

**Table 3:** The performance of SVM based model on Antifp_Main dataset developed using binary profile/pattern of peptide segments obtained from terminals.

	Parameter			Main Dataset					Validation Dataset				
	g	c	j	Sen	Spc	Acc	MCC	ROC	Sen	Spc	Acc	MCC	ROC
N5	0.5	1	2	76.07	81.66	78.86	0.58	0.86	81.16	81.25	81.21	0.62	0.87
N10	0.1	2	4	80.90	80.93	80.91	0.62	0.89	86.12	79.79	82.95	0.66	0.89
N15	0.1	1	2	82.63	82.40	82.52	0.65	0.89	86.57	81.20	83.90	0.68	0.90
C5	0.5	1	3	71.24	78.96	75.08	0.50	0.83	68.68	83.62	76.23	0.53	0.82
C10	0.1	2	2	76.99	78.67	77.84	0.56	0.86	75.36	80.43	77.92	0.56	0.84
C15	0.1	2	2	81.64	75.10	78.36	0.57	0.87	80.67	77.90	79.29	0.59	0.87
N5C5	0.1	4	2	82.02	76.59	79.20	0.59	0.87	87.55	80.49	83.88	0.68	0.90
N10C10	0.05	2	2	84.19	83.53	83.86	0.68	0.91	85.29	84.40	84.84	0.70	0.91
N15C15	0.05	1	3	85.55	84.23	84.88	0.70	0.92	85.39	83.90	84.64	0.69	0.92

### Amino Acid Composition along with Mass, Charge, and pI Value Based SVM Model

**FIGURE 4:**
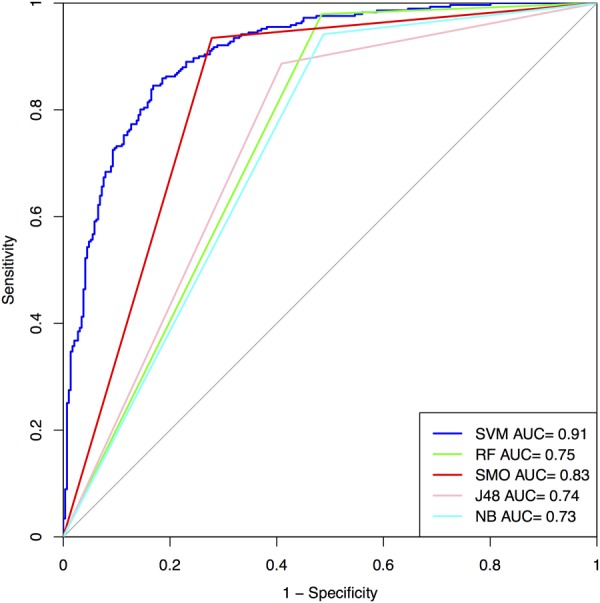
ROC curves show performance of models on Antifp_Main dataset developed using composition features along with mass, charge, and pI value.

In order to check whether the addition of mass, charge, and pI values of peptides would help in achieving better performance, we run SVM and other machine learning classifiers on the all the three datasets and developed model in order to classify AFPs from non-AFPs. We found the addition of extra three features increased the performance of MCC up to 0.01% compared to that obtained from the simple amino acid composition. We obtained accuracy of 88.78% with MCC of 0.78 on the training dataset and for validation dataset accuracy of 83.33% with MCC of 0.67 was obtained in the case of Antifp_Main (**Figure 4** and Supplementary Table S13). Similarly, results for Antifp_DS1 and Antifp_DS2 were also calculated and is given in Supplementary Tables S14, S15. However, in case of Antifp_DS2, we repeated the machine learning prediction 100 times using Scikit learn method (Pedregosa et al., 2011). This is a common practice when we handle data generated randomly from SwissProt (Bhalla et al., 2017). The mean accuracy, MCC, and ROC obtained after the process was 89.17%, 0.79 and 0.96 respectively for the training dataset and on the validation dataset mean accuracy of 90.75%, mean MCC of 0.82 and mean ROC of 0.97 was achieved. The mean standard deviation reported for accuracy, MCC and ROC were 1.01, 0.017 and 0.004 respectively. The prediction process was repeated 100 times on this particular feature because this feature was giving the best result in Antifp_DS1 and after the process was completed, we selected the dataset giving the best result and used it further for rest of the prediction process.

### Additional Benchmarking

Porto et al. (2017c) evaluate the performance of methods developed for predicting AMPs on a unique type of dataset that contains 40 designed and 38 shuffled sequences (Loose et al., 2006; Porto et al., 2017b). This dataset had compositionally similar antimicrobial and non-AMPs, the only order of residues has been changed. Existing AMP prediction methods fails on this dataset as most of these methods are based on composition. In order to repeat similar type of benchmarking for models developed in this study, we also created an Antifp_hard dataset that contains compositionally similarly AFPs and Non-AFPs (see section “Materials and Methods”). In this dataset Antifp_hard, negative sequences were compositionally quite similar to positive sequences but have entirely different activity (as explained in methods). The new dataset contained 291 AFPs and 291 non-AFPs. The accuracy obtained from the benchmarking dataset for the amino acid composition based model was decreased from 86.25% to 62.20% as compared to the validation dataset. The N15C15 binary profile feature-based model performed well as compared to composition based model. It shows the accuracy of 75.43%, as compared to 84.64% on validation dataset (**Table 4**). The results showed that model based on binary profile based feature were able to classify the sequences having compositionally similarity but different activity much better compared to composition based model.

**Table 4:** The performance of different models developed in this study and existing methods on Antifp_hard dataset contains compositionally similar peptides.

Method	Algorithm	Benchmarking Dataset						
		TP	TN	FP	FN	Sen	Spc	Acc
Composition-based model	SVM	179	183	108	112	61.51	62.89	62.20
Binary profile based model	SVM	218	92	53	48	81.95	63.45	75.43
ClassAMP	SVM	108	174	117	183	37.11	59.79	48.45
ClassAMP	Random Forest	42	221	70	249	14.43	75.94	45.18
iAMP-2L	FKNN	61	65	226	230	20.96	22.34	21.56

We also evaluate the performance of methods developed in past for predicting AFPs (i.e., ClassAMP and iAMP-2L). In case of ClassAMP, there are two types of models; one based on Random Forests and other on SVM. Positive sequences which were predicted as antifungal were labeled as true positives and not predicted as an antifungal but as other classes, were labeled as false negatives. Similarly, negative sequences predicted as other classes than antifungal were labeled as true negatives and the peptides predicted as antifungal were labeled as false positives. SVM classifier of ClassAMP showed the accuracy of 48.45% whereas random forest classifier of the same method showed the accuracy of 45.18% (**Table 4**).

In case of iAMPL-2L, any AFP predicted as antifungal, even if it is predicted to belong to any other class were labeled as true positive. Rest positive peptides are labeled as false negatives. Similarly, any negative dataset peptide predicted as non-antifungal is labeled as true negative and rest negative peptides are labeled as false positives. The iAMPL-2L method showed the accuracy of 21.56% (**Table 4**).

**FIGURE 5:**
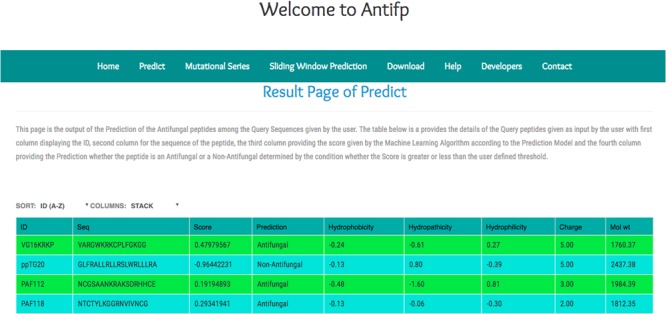
Screenshot of the “Antifp” predict page showing the result of the sequences taken in case study.

In addition, we compiled the sequences from the different study and submitted them to our server to check the performance of our model. We observed that our binary profile based model was able to predict three peptides correctly out of four peptides. Here, we are providing the screenshot of the result page of the binary profile based model below (**Figure 5**).

### Implementation of Web Server

**FIGURE 6:**
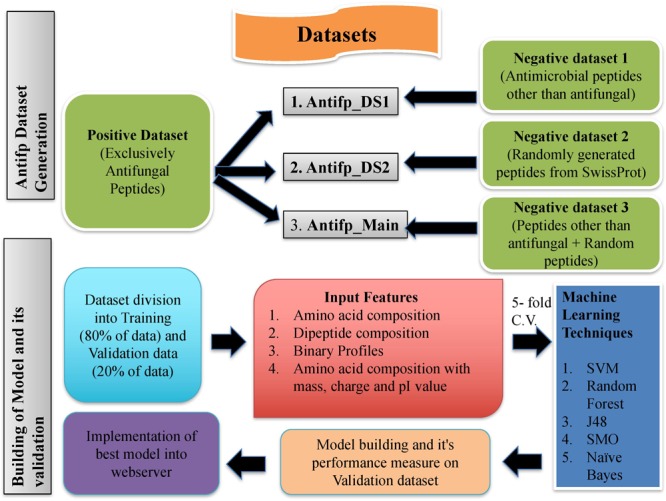
Schematic representation of procedure used to create datasets and building models in this study.

We have tried to develop a class-specific prediction web server for the prediction of AFPs, exclusively. Thus, to assist the scientific community, we have implemented our three best models trained on three different datasets. An overall prediction approach of Antifp is shown in **Figure 6**. ‘Model 1,’ developed on the Antifp_DS1 N15C15 binary profile feature will be useful for those users who wish to check whether their peptides possess exclusively antifungal activity and no other antimicrobial activity. Second model ‘Model 2,’ developed on the Antifp_DS2, found to performing best on N15C15 binary profile feature will be useful for those users who do not have any previous knowledge of their peptide and want to check whether their peptide possess antifungal property or not. This peptide may have other antimicrobial properties too. Finally, third model, ‘Model 3’ developed on the Antifp_Main and performing best on the same feature as of ‘Model 1,’ will be helpful for those users who do not have previous knowledge of their peptide and want the exclusively antifungal property. All the above three mentioned models were implemented in a user-friendly web server ‘Antifp’ by the name “Antifp_DS1_binary_model1,” “Antifp_DS1_binary_model 2,” and “Antifp_Main_binary_model 3,” respectively.

#### Predict Page

This page provides the option to check whether the submitted query is AFP or non-AFP. This page takes the sequence in FASTA format as an input and displays the prediction as output along with the prediction score at the chosen threshold cut-off value by the user. Here, a user can submit either single sequence or number of sequences at a time. In addition, the server also provides facility to calculate important physiochemical properties of the submitted query sequence.

#### Mutational Series Page

‘Antifp’ web server along with the facility to predict peptides, as AFPs or non-AFPs also provides an opportunity to design analogs with enhanced antifungal properties. All possible mutant of given peptides with the single mutation can be obtained by giving single letter code of peptide sequence (no FASTA format required) as input to the design module of the web server. For all mutant peptides, the server will give a result, which constitutes an SVM score and prediction status of AFPs and non-AFPs according to threshold cut-off selected by the user. As a provision for selecting the threshold is provided in the server, the user is suggested to select a higher value to get high specificity. Therefore, the feature as mention above will be useful for users in designing new and highly effective AFP analogs. In this page, original input peptide will be shown along with all generated analogs, and sorting option is also provided in the table, which can be used to sort the peptide analogs based on preferred properties and ultimately to choose the best peptide analog.

#### Sliding Window Prediction Page

Another information tool is the protein-scanning tool for the discovery of putative AFPs. Here, a user may give the protein sequence as input and select the window length to generate overlapping peptides of window length, where each of the peptides will be ranked according to its score. This will help in determining the possible regions in protein sequence, which could be antifungal. We have also provided a download module from where the user can download the dataset used in this study. Antifp is freely accessible at http://webs.iiitd.edu.in/raghava/antifp.

### Antifp Standalone Software

The standalone software was also developed in order to let the users predict and analyze the peptide sequences even in the absence of Internet. The standalone was developed for Linux, Mac as well as Windows 64-bit operating systems. The software was developed using Python (v2.7.11) and wxPython (v.3.0.0) platform. The standalone is implemented with our best model which takes FASTA sequence of peptides as input. It provides comparable results to that of the online server. The software is bundled with all the required files and libraries in the zip file format and can be downloaded freely from the ‘Download’ menu of the online server ‘Antifp’^1^.

### Anitfp Mobile App

We have also developed a mobile app of Antifp for the android users where the user can use this service by downloading and installing the app. The app was developed using Python (v2.7.11) and kivy (v1.9.2). The app is implemented with our best model which takes both sequences as well as the file as input. The minimum length of the sequence should be 15 or more. The app consists of the following module:

**(i) Predict:** This module will be helpful in predicting the antifungal property of the given peptide sequence as input. A user can either give raw peptide sequence or multiple sequences in FASTA format.
**(ii) Mutational Series:** This module will produce mutant analogs of the given peptide sequence and predict the antifungal property for each of them.
**(iii) Sliding Window Prediction:** This module scans the protein sequence given as an input and will create overlapping peptides of the particular window size given by the user. The module will also provide result whether the generated peptides are antifungal or not.

The ‘Antifp Mobile App’ is provided in the standard “apk” file format and can be freely downloaded from the ‘Download’ menu of the online server ‘Antifp’^1^.

## Discussion

Due to pronounced therapeutic applications of AFPs, identification and designing of the novel and highly efficient AFPs is need of the hour but it is a very tedious and time-consuming task for the biologists. One has to scan the whole protein in overlapping windows patterns, and every peptide has to be tested for the possible antifungal activity. An *in silico* method, which can predict in advance whether a peptide sequence can be AFP or not, would definitely help experimental biologists for a speedy screening of AFPs before synthesis and thus, fasten the AFP based research. Development of a computational method for AFP prediction is challenging due to various reasons since (i) AFPs have a lot of flexibility in size (4–100 amino acids) and fixed length pattern is required as input by machine learning methods to develop a model (ii) due to lack of experimentally validated AFPs. Till date, a very limited study has been done in this area and currently; there are no web services available exclusively for prediction and designing of AFPs. In the last few years, a vast number of AMPs have been reported which might act as AFPs and this vast amount of data inspired us to develop a computational method on the larger dataset of 1459 AFPs. In order to discriminate AFPs from non-AFPs with higher precision, we have developed SVM models based on features like amino acid composition, dipeptide composition, amino acid composition along with mass, charge and pI value, binary profile, N and C-terminal residue hybrid. The performance of the models developed was found to be quite impressive when features like amino acid composition, amino acid composition along with mass, charge, and pI value and dipeptide composition were used as input. We have also developed SVM models based on a binary profile of patterns, which integrates information on both amino acid order and its composition. It was observed that this feature performed better than the composition-based model. In Antifp_DS2, hybrid of N15C15 binary profile feature outperformed other features based model.

Discriminating two sequences with high identity but different activity is a challenging task for most of the prediction methods. To address this issue, we calculated the euclidean distance between our positive and negative peptides and selected the negative peptides with minimum distance. We tested the performance of our composition based model as well as N15C15 binary profile based model and observed that composition model didn’t perform well in discriminating two sequences very accurately. However, our binary profile based model was able to discriminate the two sequences with good accuracy, suggesting that binary profile feature can be used in discriminating such sequences where sequences are very similar to each other but possess different activity. We also tested the performance of two previous methods ClassAMP and iAMP-2L on this dataset, where they failed to discriminate the two sequences. In comparison to the above-mentioned methods, our method performed better.

Preliminary composition analysis has shown that AFPs are rich in cationic residues like C, G, H, K, and R in comparison to non-AFPs. Presence of the positively charged residues allows the peptide to interact with negatively charged membrane and carry out cell lysis. Positional residue preference studies showed that residues like R, V, and K are mostly preferred at N-terminal positions whereas residues like C and H are highly preferred at C-terminus in AFPs. In addition, we have also looked out for motifs, which could potentially be a part of AFPs using MERCI software. We were able to find out various motifs. Furthermore, to help biologists and serve scientific community, best models are implemented in a user-friendly web server ‘Antifp,’ mobile app and standalone where a user can predict whether their peptide or series of peptides are AFP or non-AFP in nature.

Antifp, though has certain limitations like the method does not consider modifications (e.g., post-translational modifications) and other topological aspects during model development. Secondly, our method cannot predict in advance that putative peptides designed using the design tool, will show broad-spectrum activities or not. However, our method is likely to help biologists in designing a better peptide-based drug.

## Author Contributions

PA and KC collected the data and created the datasets. PA, SB, and KC analyzed the results, developed the computer programs, implemented SVM, and other techniques. RK, SB, and PA developed the front-end user interface and created the back-end server. MS developed the standalone and mobile apps. PA, RK, and KC wrote the manuscript. GPSR conceived the idea and coordinated the project.

## Conflict of Interest Statement

The authors declare that the research was conducted in the absence of any commercial or financial relationships that could be construed as a potential conflict of interest.

## Footnotes

1 http://webs.iiitd.edu.in/raghava/antifp/algo.php
