# Supplementary material for: In Silico Approach for Prediction of Antifungal Peptides
Source: Front Microbiol. 2018 Feb 26;9:323. doi: 10.3389/fmicb.2018.00323 (PMC5834480; doi:10.3389/fmicb.2018.00323)
Supplement: Supplementary file 1 [file Data_Sheet_1.DOC]

**In silico approach for prediction of antifungal peptides**

**Piyush Agrawal^1#^, Sherry Bhalla^1#^, Kumardeep Chaudhary^1^, Rajesh Kumar^1^, Meenu Sharma^1^, Gajendra P.S. Raghava*^,1,2^**

1. CSIR-Institute of Microbial Technology, Chandigarh 160036, India.

2. Center for Computational Biology, Indraprastha Institute of Information Technology, New Delhi 110020, India.

^#^Authors Contributed Equally

**^*^ Corresponding author**

Professor of Computation Biology, Indraprastha Institute of Information Technology (IIIT Delhi), Okhla Phase III, New Delhi-110020, India. Phone: +91-172-26907444; Fax: +91-172-26907410

email: [raghava@iiitd.ac.in](mailto:raghava@iiitd.ac.in)

**Supplementary Information**

**MOTIFS**

**Table S1. Exclusive motifs of Antifp_Main.**

| **Sr. No.** | **Positive Motifs** | **Negative Motifs** |
| --- | --- | --- |
| 1 | CFCT | CGNTK |
| 2 | RCFC | CGNTKH |
| 3 | NCAS | CGNTKHC |
| 4 | RCFCT | CGNTKHCD |
| 5 | CASV | GNTK |
| 6 | NCASV | GNTKH |
| 7 | RTCE | GNTKHC |
| 8 | CASVC | GNTKHCD |
| 9 | NCASVC | NTKH |
| 10 | RRCFC | NTKHC |
| 11 | SHKF | NTKHCD |
| 12 | SVCQ | - |
| 13 | TCES | - |

**Table S2. Exclusive motifs of Antifp_DS1.**

| **Sr. No.** | **Positive Motifs** | **Negative Motifs** |
| --- | --- | --- |
| 1 | RCFC | CGNTK |
| 2 | NCAS | CGNTKH |
| 3 | RCFCT | CGNTKHC |
| 4 | CASV | CGNTKHCD |
| 5 | NCASV | GNTK |
| 6 | RTCE | GNTKH |
| 7 | CASVC | GNTKHC |
| 8 | NCASVC | GNTKHCD |
| 9 | RRCFC | NTKH |
| 10 | SHKF | NTKHC |
| 11 | SVCQ | NTKHCD |
| 12 | TCES | - |

**Table S3. Exclusive motifs of Antifp_DS2.**

| **Sr. No.** | **Positive Motifs** | **Negative Motifs** |
| --- | --- | --- |
| 1 | GSCN | DFA |
| 2 | SCNY | GTI |
| 3 | CNYV | EAF |
| 4 | GSCNY | GMA |
| 5 | HGSC | FDA |
| 6 | HGSCN | VDF |
| 7 | SCNYV | INP |
| 8 | CNYVF | IYQ |
| 9 | GSCNYV | DIT |
| 10 | HGSCNY | PFA |

**Table S4. The performance of different machine learning techniques based models on Antifp_DS1 developed using amino acid composition of peptides.**

|  | **Parameters** | **Main Dataset** | | | | | **Validation Dataset** | | | | |
| --- | --- | --- | --- | --- | --- | --- | --- | --- | --- | --- | --- |
|  |  | **Sen** | **Spc** | **Acc** | **MCC** | **ROC** | **Sen** | **Spc** | **Acc** | **MCC** | **ROC** |
| SVM | g=0.01, c=2, j=2 | 86.90 | 85.62 | 86.26 | 0.73 | 0.93 | 84.54 | 87.29 | 85.91 | 0.72 | 0.93 |
| Random Forest | Ntree = 350 | 85.45 | 84.16 | 84.80 | 0.70 | 0.93 | 81.10 | 79.04 | 80.07 | 0.60 | 0.87 |
| SMO | g=0.001, c=5 | 86.56 | 80.57 | 83.56 | 0.67 | 0.83 | 82.13 | 83.85 | 82.99 | 0.66 | 0.82 |
| J48 | c=0.25, m= 3 | 77.05 | 77.23 | 77.14 | 0.54 | 0.78 | 72.16 | 75.60 | 73.88 | 0.48 | 0.74 |
| Naïve Bayes | Default | 74.40 | 63.53 | 68.96 | 0.38 | 0.72 | 67.70 | 66.67 | 67.18 | 0.34 | 0.72 |

**Table S5. The performance of different machine learning techniques based models on Antifp_DS2 developed using amino acid composition of peptides..**

|  | **Parameters** | **Main Dataset** | | | | | **Validation Dataset** | | | | |
| --- | --- | --- | --- | --- | --- | --- | --- | --- | --- | --- | --- |
|  |  | **Sen** | **Spc** | **Acc** | **MCC** | **ROC** | **Sen** | **Spc** | **Acc** | **MCC** | **ROC** |
| SVM | g= 0.005, c= 5, j=1 | 93.24 | 92.38 | 92.81 | 0.86 | 0.97 | 90.72 | 90.03 | 90.38 | 0.81 | 0.96 |
| Random Forest | Ntree = 50 | 91.95 | 91.35 | 91.65 | 0.83 | 0.97 | 87.97 | 86.60 | 87.29 | 0.75 | 0.93 |
| SMO | g=0.001, c=3 | 90.24 | 91.44 | 90.84 | 0.82 | 0.90 | 91.07 | 90.38 | 90.72 | 0.81 | 0.90 |
| J48 | c=0.2, m=6 | 88.87 | 87.07 | 87.97 | 0.76 | 0.91 | 87.97 | 85.57 | 86.77 | 0.74 | 0.90 |
| Naive Bayes | Default | 84.42 | 87.24 | 85.83 | 0.72 | 0.91 | 83.16 | 85.57 | 84.36 | 0.69 | 0.90 |

**Table S6*. The performance of SVM based models on Antifp_Main dataset, where models were developed using amino acid composition of part of peptides.**

|  | **Parameters** | | | **Main dataset** | | | | **Validation dataset** | | | |
| --- | --- | --- | --- | --- | --- | --- | --- | --- | --- | --- | --- |
|  | **g** | **c** | **j** | **Sen** | **Spc** | **Acc** | **MCC** | **Sen** | **Spc** | **Acc** | **MCC** |
| N5 | 0.0005 | 2 | 2 | 75.62 | 73.08 | 74.35 | 0.49 | 77.17 | 75.35 | 76.24 | 0.53 |
| N10 | 0.005 | 1 | 2 | 80.18 | 75.76 | 77.96 | 0.56 | 82.56 | 76.60 | 79.57 | 0.59 |
| N15 | 0.01 | 1 | 2 | 79.87 | 75.78 | 77.81 | 0.56 | 79.85 | 81.95 | 80.90 | 0.62 |
| C5 | 0.001 | 1 | 1 | 71.68 | 72.93 | 72.30 | 0.45 | 68.68 | 75.26 | 72.01 | 0.44 |
| C10 | 0.005 | 1 | 2 | 79.53 | 76.08 | 77.79 | 0.56 | 78.62 | 77.94 | 78.28 | 0.57 |
| C15 | 0.005 | 1 | 1 | 79.07 | 80.70 | 79.89 | 0.60 | 79.55 | 77.15 | 78.36 | 0.57 |
| N5C5 | 0.0005 | 2 | 2 | 80.26 | 77.77 | 78.96 | 0.58 | 83.02 | 78.05 | 80.43 | 0.61 |
| N10C10 | 0.001 | 2 | 1 | 84.47 | 81.05 | 82.75 | 0.66 | 86.76 | 81.56 | 84.12 | 0.68 |
| N15C15 | 0.001 | 2 | 1 | 86.90 | 86.62 | 86.76 | 0.74 | 87.27 | 86.89 | 87.08 | 0.74 |

* ROC values are already provided in the Figure 3 in the main manuscript.

**Table S7. The performance of SVM based models on Antifp_DS1, where models were developed using amino acid composition of part of peptides.**

|  | **Parameters** | | | **Main Dataset** | | | | | **Validation Dataset** | | | | |
| --- | --- | --- | --- | --- | --- | --- | --- | --- | --- | --- | --- | --- | --- |
|  | **g** | **c** | **j** | **Sen** | **Spc** | **Acc** | **MCC** | **ROC** | **Sen** | **Spc** | **Acc** | **MCC** | **ROC** |
| N5 | 0.001 | 7 | 2 | 67.39 | 81.63 | 74.51 | 0.50 | 0.81 | 70.36 | 85.51 | 77.98 | 0.57 | 0.82 |
| N10 | 0.001 | 15 | 1 | 80.16 | 79.55 | 79.86 | 0.60 | 0.86 | 75.44 | 77.22 | 76.33 | 0.53 | 0.82 |
| N15 | 0.005 | 1 | 2 | 82.47 | 77.32 | 79.89 | 0.60 | 0.89 | 85.50 | 81.68 | 83.62 | 0.67 | 0.91 |
| C5 | 0.001 | 1 | 1 | 69.37 | 80.37 | 74.77 | 0.50 | 0.82 | 60.79 | 80.15 | 70.36 | 0.42 | 0.79 |
| C10 | 0.001 | 5 | 2 | 79.21 | 76.38 | 77.79 | 0.56 | 0.85 | 73.21 | 76.98 | 75.09 | 0.50 | 0.82 |
| C15 | 0.005 | 1 | 2 | 81.66 | 76.83 | 79.27 | 0.59 | 0.88 | 79.70 | 78.24 | 78.98 | 0.58 | 0.87 |
| N5C5 | 0.0005 | 1 | 1 | 80.14 | 77.28 | 78.69 | 0.57 | 0.86 | 77.49 | 79.27 | 78.39 | 0.57 | 0.88 |
| N10C10 | 0.001 | 2 | 1 | 86.21 | 80.40 | 83.33 | 0.67 | 0.92 | 84.64 | 87.96 | 86.28 | 0.73 | 0.93 |
| N15C15 | 0.001 | 3 | 2 | 85.71 | 84.02 | 84.88 | 0.70 | 0.92 | 81.27 | 87.36 | 84.28 | 0.69 | 0.93 |

**Table S8. The performance of SVM based models on Antifp_DS2, where models were developed using amino acid composition of part of peptides.**

|  | **Parameters** | | | **Main Dataset** | | | | | **Validation Dataset** | | | | |
| --- | --- | --- | --- | --- | --- | --- | --- | --- | --- | --- | --- | --- | --- |
|  | **g** | **c** | **j** | **Sen** | **Spc** | **Acc** | **MCC** | **ROC** | **Sen** | **Spc** | **Acc** | **MCC** | **ROC** |
| N5 | 0.001 | 1 | 2 | 78.80 | 78.76 | 78.78 | 0.58 | 0.85 | 80.34 | 77.35 | 78.56 | 0.58 | 0.85 |
| N10 | 0.001 | 2 | 1 | 82.44 | 79.18 | 80.82 | 0.62 | 0.87 | 84.75 | 76.16 | 80.46 | 0.61 | 0.89 |
| N15 | 0.005 | 3 | 1 | 83.80 | 81.78 | 82.78 | 0.66 | 0.90 | 86.62 | 82.77 | 84.70 | 0.69 | 0.91 |
| C5 | 0.0001 | 5 | 2 | 76.18 | 73.47 | 74.83 | 0.50 | 0.81 | 75.17 | 74.22 | 74.70 | 0.49 | 0.82 |
| C10 | 0.0005 | 2 | 2 | 82.00 | 79.71 | 80.87 | 0.62 | 0.87 | 81.21 | 76.87 | 79.04 | 0.58 | 0.86 |
| C15 | 0.005 | 2 | 1 | 84.92 | 81.68 | 83.29 | 0.67 | 0.90 | 81.41 | 80.90 | 81.16 | 0.62 | 0.88 |
| N5C5 | 0.0005 | 1 | 5 | 85.74 | 82.72 | 84.13 | 0.68 | 0.91 | 87.64 | 81.18 | 84.30 | 0.69 | 0.92 |
| N10C10 | 0.001 | 1 | 3 | 89.92 | 85.70 | 87.77 | 0.76 | 0.94 | 91.94 | 85.41 | 88.63 | 0.77 | 0.94 |
| N15C15 | 0.001 | 1 | 5 | 92.13 | 88.71 | 90.39 | 0.81 | 0.95 | 91.01 | 86.89 | 88.95 | 0.78 | 0.95 |

**Table S9. The performance of SVM based models on Antifp_DS1, where models were developed using dipeptide composition of whole peptide and part of peptides.**

|  | **Parameters** | | | **Main Dataset** | | | | | **Validation Dataset** | | | | |
| --- | --- | --- | --- | --- | --- | --- | --- | --- | --- | --- | --- | --- | --- |
|  | **g** | **c** | **j** | **Sen** | **Spc** | **Acc** | **MCC** | **ROC** | **Sen** | **Spc** | **Acc** | **MCC** | **ROC** |
| DPC | 0.005 | 4 | 2 | 87.24 | 87.16 | 87.20 | 0.74 | 0.94 | 88.32 | 84.88 | 86.60 | 0.73 | 0.94 |
| N5 | 0.0005 | 2 | 1 | 80.34 | 74.12 | 77.23 | 0.55 | 0.87 | 83.57 | 69.96 | 76.73 | 0.54 | 0.87 |
| N10 | 0.001 | 2 | 2 | 80.99 | 80.54 | 80.76 | 0.62 | 0.90 | 81.75 | 84.34 | 83.04 | 0.66 | 0.91 |
| N15 | 0.001 | 2 | 1 | 84.49 | 79.83 | 82.16 | 0.64 | 0.89 | 84.01 | 85.11 | 84.56 | 0.69 | 0.92 |
| C5 | 0.001 | 1 | 3 | 74.32 | 79.07 | 76.65 | 0.53 | 0.85 | 70.14 | 79.41 | 74.73 | 0.50 | 0.83 |
| C10 | 0.0005 | 8 | 1 | 81.96 | 76.20 | 79.06 | 0.58 | 0.87 | 80.36 | 74.10 | 77.24 | 0.55 | 0.86 |
| C15 | 0.001 | 2 | 3 | 81.18 | 79.36 | 80.28 | 0.61 | 0.89 | 80.83 | 75.19 | 78.03 | 0.56 | 0.87 |
| N5C5 | 0.0001 | 15 | 1 | 78.25 | 77.38 | 77.81 | 0.56 | 0.85 | 76.01 | 83.27 | 79.67 | 0.59 | 0.87 |
| N10C10 | 0.0005 | 1 | 1 | 86.49 | 78.97 | 82.77 | 0.66 | 0.91 | 85.00 | 81.75 | 83.39 | 0.67 | 0.91 |
| N15C15 | 0.001 | 4 | 1 | 85.23 | 84.42 | 84.83 | 0.70 | 0.92 | 84.64 | 83.91 | 84.28 | 0.69 | 0.93 |

**Table S10. The performance of SVM based models on Antifp_DS2, where models were developed using dipeptide composition of whole peptide and part of peptides.**

|  | **Parameters** | | | **Main Dataset** | | | | | **Validation Dataset** | | | | |
| --- | --- | --- | --- | --- | --- | --- | --- | --- | --- | --- | --- | --- | --- |
|  | **g** | **c** | **j** | **Sen** | **Spc** | **Acc** | **MCC** | **ROC** | **Sen** | **Spc** | **Acc** | **MCC** | **ROC** |
| DPC | 0.001 | 4 | 3 | 91.95 | 91.78 | 91.87 | 0.84 | 0.96 | 92.78 | 91.41 | 92.10 | 0.84 | 0.96 |
| N5 | 0.0001 | 15 | 3 | 81.72 | 80.31 | 81.01 | 0.62 | 0.87 | 82.76 | 78.05 | 80.42 | 0.61 | 0.87 |
| N10 | 0.0005 | 3 | 2 | 83.06 | 81.41 | 82.24 | 0.64 | 0.90 | 87.94 | 85.05 | 86.50 | 0.73 | 0.94 |
| N15 | 0.001 | 2 | 3 | 85.10 | 83.16 | 84.13 | 0.68 | 0.92 | 89.22 | 85.39 | 87.31 | 0.75 | 0.94 |
| C5 | 0.001 | 1 | 2 | 73.77 | 78.73 | 76.25 | 0.53 | 0.83 | 76.21 | 82.58 | 79.38 | 0.59 | 0.85 |
| C10 | 0.001 | 3 | 1 | 83.23 | 79.27 | 81.27 | 0.63 | 0.89 | 79.43 | 81.14 | 80.28 | 0.61 | 0.89 |
| C15 | 0.001 | 2 | 1 | 84.73 | 82.89 | 83.81 | 0.68 | 0.91 | 82.16 | 83.52 | 82.84 | 0.66 | 0.90 |
| N5C5 | 0.0001 | 2 | 4 | 84.38 | 83.58 | 83.95 | 0.68 | 0.91 | 85.02 | 81.53 | 83.21 | 0.67 | 0.92 |
| N10C10 | 0.0005 | 2 | 2 | 87.70 | 86.15 | 86.91 | 0.74 | 0.94 | 90.11 | 87.90 | 88.99 | 0.78 | 0.95 |
| N15C15 | 0.001 | 2 | 2 | 90.12 | 89.45 | 89.78 | 0.80 | 0.95 | 90.64 | 89.14 | 89.89 | 0.80 | 0.95 |

**Table S11. The performance of SVM based model on Antifp_DS1 developed using binary profile/pattern of peptide segments obtained from terminals.**

|  | **Parameters** | | | **Main Dataset** | | | | | **Validation Dataset** | | | | |
| --- | --- | --- | --- | --- | --- | --- | --- | --- | --- | --- | --- | --- | --- |
|  | **g** | **c** | **j** | **Sen** | **Spc** | **Acc** | **MCC** | **ROC** | **Sen** | **Spc** | **Acc** | **MCC** | **ROC** |
| N5 | 0.5 | 1 | 3 | 82.88 | 76.11 | 79.49 | 0.59 | 0.89 | 81.43 | 75.62 | 78.51 | 0.57 | 0.88 |
| N10 | 0.05 | 6 | 4 | 84.00 | 79.91 | 81.93 | 0.64 | 0.90 | 82.11 | 76.16 | 79.15 | 0.58 | 0.87 |
| N15 | 0.1 | 1 | 3 | 83.72 | 79.73 | 81.73 | 0.64 | 0.91 | 82.53 | 80.53 | 81.54 | 0.63 | 0.89 |
| C5 | 0.5 | 1 | 2 | 76.94 | 75.89 | 76.42 | 0.53 | 0.86 | 70.86 | 76.47 | 73.64 | 0.47 | 0.83 |
| C10 | 0.05 | 6 | 4 | 82.78 | 74.03 | 78.38 | 0.57 | 0.88 | 76.07 | 73.74 | 74.91 | 0.50 | 0.84 |
| C15 | 0.05 | 3 | 3 | 79.75 | 79.26 | 79.51 | 0.59 | 0.88 | 75.19 | 77.10 | 76.14 | 0.52 | 0.85 |
| N5C5 | 0.1 | 2 | 2 | 81.43 | 78.54 | 79.97 | 0.60 | 0.88 | 80.07 | 76.73 | 78.39 | 0.57 | 0.87 |
| N10C10 | 0.1 | 1 | 3 | 84.90 | 80.98 | 82.95 | 0.66 | 0.92 | 83.21 | 78.10 | 80.69 | 0.61 | 0.90 |
| N15C15 | 0.1 | 4 | 2 | 86.49 | 82.32 | 84.44 | 0.69 | 0.92 | 82.02 | 81.23 | 81.63 | 0.63 | 0.92 |

**Table S12. The performance of SVM based model on Antifp_DS2 developed using binary profile/pattern of peptide segments obtained from terminals.**

|  | **Parameters** | | | **Main Dataset** | | | | | **Validation Dataset** | | | | |
| --- | --- | --- | --- | --- | --- | --- | --- | --- | --- | --- | --- | --- | --- |
|  | **g** | **c** | **j** | **Sen** | **Spc** | **Acc** | **MCC** | **ROC** | **Sen** | **Spc** | **Acc** | **MCC** | **ROC** |
| N5 | 0.5 | 1 | 2 | 80.26 | 88.05 | 84.15 | 0.69 | 0.90 | 86.55 | 87.11 | 86.83 | 0.74 | 0.92 |
| N10 | 0.05 | 5 | 2 | 86.13 | 85.97 | 86.05 | 0.72 | 0.91 | 88.30 | 87.90 | 88.10 | 0.76 | 0.93 |
| N15 | 0.05 | 5 | 1 | 88.18 | 86.40 | 87.29 | 0.75 | 0.93 | 88.85 | 86.52 | 87.69 | 0.75 | 0.94 |
| C5 | 0.05 | 10 | 1 | 77.99 | 75.71 | 76.85 | 0.54 | 0.84 | 75.86 | 79.44 | 77.64 | 0.55 | 0.84 |
| C10 | 0.1 | 1 | 4 | 83.06 | 78.37 | 80.74 | 0.62 | 0.89 | 80.14 | 83.63 | 81.88 | 0.64 | 0.88 |
| C15 | 0.1 | 2 | 2 | 84.82 | 83.81 | 84.32 | 0.69 | 0.92 | 85.50 | 86.14 | 85.82 | 0.72 | 0.90 |
| N5C5 | 0.1 | 1 | 4 | 87.11 | 85.55 | 86.28 | 0.73 | 0.93 | 89.89 | 89.90 | 89.89 | 0.80 | 0.94 |
| N10C10 | 0.05 | 1 | 3 | 90.56 | 90.17 | 90.36 | 0.81 | 0.95 | 90.84 | 92.88 | 91.88 | 0.84 | 0.96 |
| N15C15 | 0.05 | 2 | 1 | 92.51 | 92.14 | 92.32 | 0.85 | 0.97 | 92.51 | 92.88 | 92.70 | 0.85 | 0.97 |

**Table S13*. Performance of different machine learning methods on amino acid composition along with mass, charge and pI value as a feature on Antifp_Main.**

|  | **Parameters** | **Main dataset** | | | | **Validation dataset** | | | |
| --- | --- | --- | --- | --- | --- | --- | --- | --- | --- |
|  |  | **Sen** | **Spc** | **Acc** | **MCC** | **Sen** | **Spc** | **Acc** | **MCC** |
| SVM | g=0.005, c=4, j=2 | 88.78 | 88.78 | 88.78 | 0.78 | 84.88 | 81.79 | 83.33 | 0.67 |
| Random Forest | Ntree = 300 | 88.36 | 87.59 | 87.97 | 0.76 | 97.94 | 51.55 | 74.74 | 0.56 |
| SMO | g=0.001, c=5 | 89.38 | 84.50 | 86.94 | 0.74 | 93.47 | 72.16 | 82.82 | 0.67 |
| J48 | c=0.4, m=4 | 81.85 | 80.14 | 80.99 | 0.62 | 88.66 | 59.11 | 73.88 | 0.50 |
| Naive Bayes | Default | 80.99 | 80.05 | 80.52 | 0.61 | 92.78 | 55.33 | 74.05 | 0.52 |

* ROC values are already provided in the Figure 4 in the main manuscript.

**Table S14. Performance of different machine learning methods on amino acid composition along with mass, charge and pI value as feature on Antifp_DS1.**

|  | **Parameters** | **Main Dataset** | | | | | **Validation Dataset** | | | | |
| --- | --- | --- | --- | --- | --- | --- | --- | --- | --- | --- | --- |
|  |  | **Sen** | **Spc** | **Acc** | **MCC** | **ROC** | **Sen** | **Spc** | **Acc** | **MCC** | **ROC** |
| SVM | g=0.005, c=7, j=5 | 88.10 | 87.41 | 87.76 | 0.76 | 0.95 | 73.88 | 89.35 | 81.62 | 0.64 | 0.89 |
| Random Forest | Ntree = 250 | 87.84 | 87.76 | 87.80 | 0.76 | 0.94 | 72.85 | 60.14 | 66.49 | 0.33 | 0.73 |
| SMO | g=0.001, c=4 | 90.58 | 83.56 | 87.07 | 0.74 | 0.87 | 87.29 | 81.79 | 84.54 | 0.69 | 0.84 |
| J48 | c=0.25, m=7 | 79.02 | 78.25 | 78.64 | 0.57 | 0.84 | 74.23 | 52.58 | 63.40 | 0.27 | 0.65 |
| Naive Bayes | Default | 75.34 | 66.01 | 70.68 | 0.42 | 0.76 | 83.16 | 54.30 | 68.73 | 0.39 | 0.75 |

**Table S15. Performance of different machine learning methods on amino acid composition along with mass, charge and pI value as feature on Antifp_DS2.**

|  | **Parameters** | **Main Dataset** | | | | | **Validation Dataset** | | | | |
| --- | --- | --- | --- | --- | --- | --- | --- | --- | --- | --- | --- |
|  |  | **Sen** | **Spc** | **Acc** | **MCC** | **ROC** | **Sen** | **Spc** | **Acc** | **MCC** | **ROC** |
| SVM (Scikit)* | g=0.001, c=2 | 86.99 | 91.35 | 89.17 | 0.79 | 0.96 | 90.71 | 90.79 | 90.75 | 0.82 | 0.97 |
| Random Forest | Ntree = 20 | 92.29 | 91.78 | 92.04 | 0.84 | 0.97 | 94.50 | 80.41 | 87.46 | 0.76 | 0.95 |
| SMO | g=0.001, c=5 | 92.89 | 93.92 | 93.41 | 0.87 | 0.93 | 93.81 | 88.66 | 91.24 | 0.83 | 0.91 |
| J48 | c=0.25, m=1 | 90.92 | 89.64 | 90.28 | 0.81 | 0.88 | 91.07 | 84.54 | 87.80 | 0.76 | 0.84 |
| Naive Bayes | Default | 89.90 | 88.53 | 89.21 | 0.78 | 0.94 | 93.47 | 82.82 | 88.14 | 0.77 | 0.93 |

*** Mean value obtained after repeating SVM 100 time is provided**

**Figure S1. Comparison of percent average amino acid composition of the AFPs and non-AFPs of (A) Antifp_DS1 and (B) Antifp_DS2.**

**
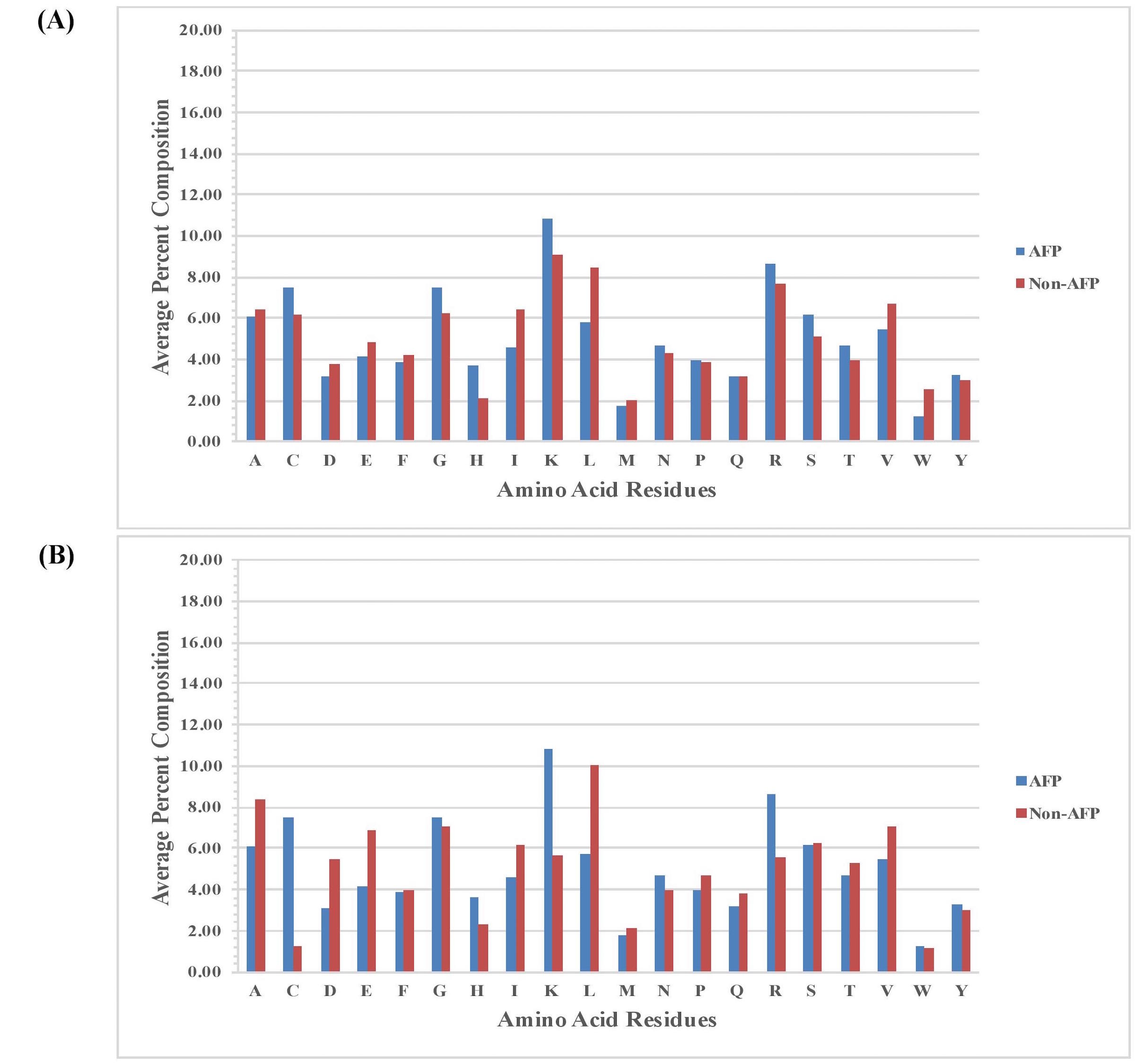
**

**Figure S2. Comparison of percent average amino acid composition of the (A) first 15 N-terminal and (B) first 15 C-terminal AFPs and non-AFPs of Antifp_DS1.**

**
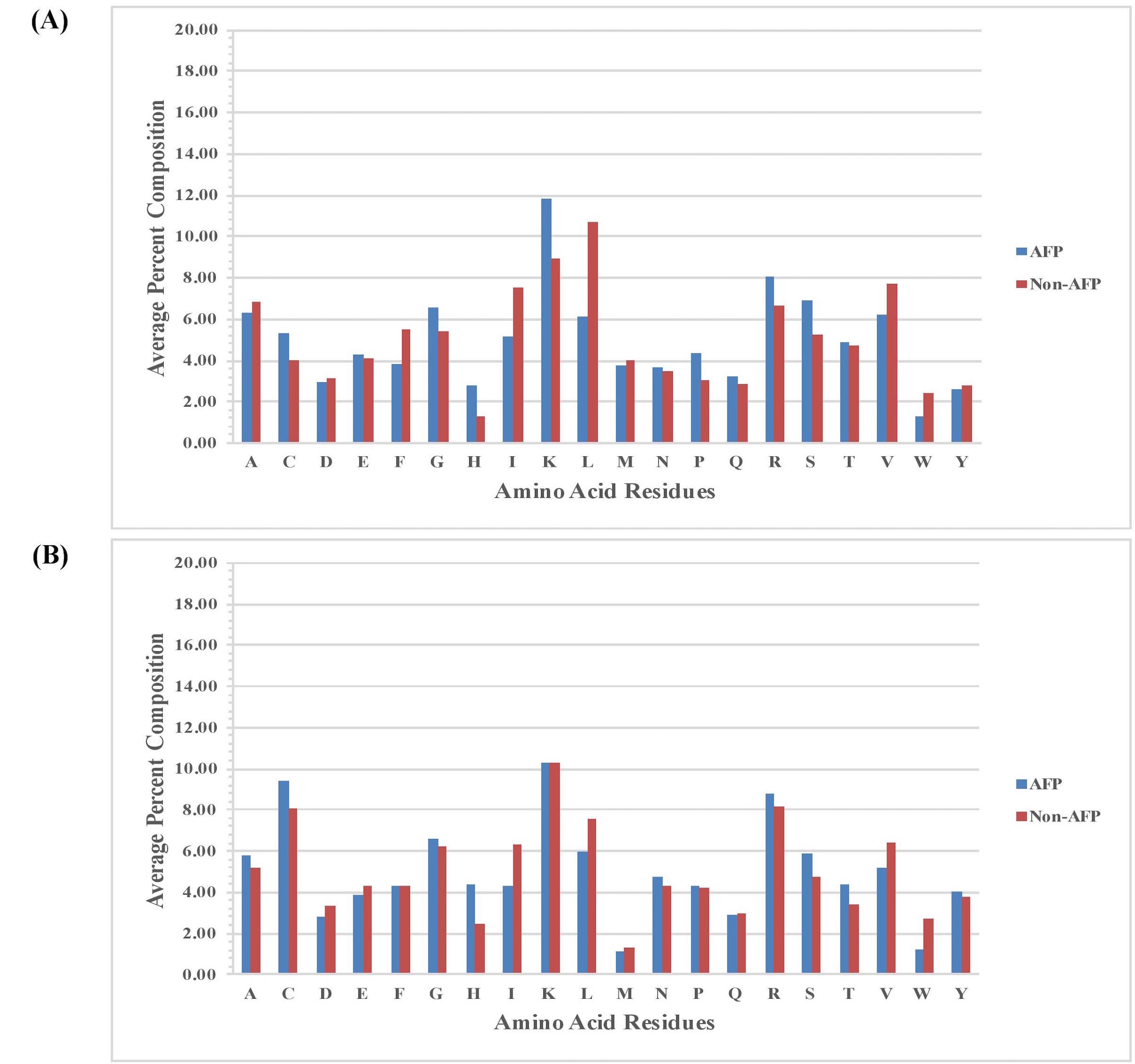
**

**Figure S3. Comparison of percent average amino acid composition of the (A) first 15 N-terminal and (B) first 15 C-terminal AFPs and non-AFPs of Antifp_DS2.**

**
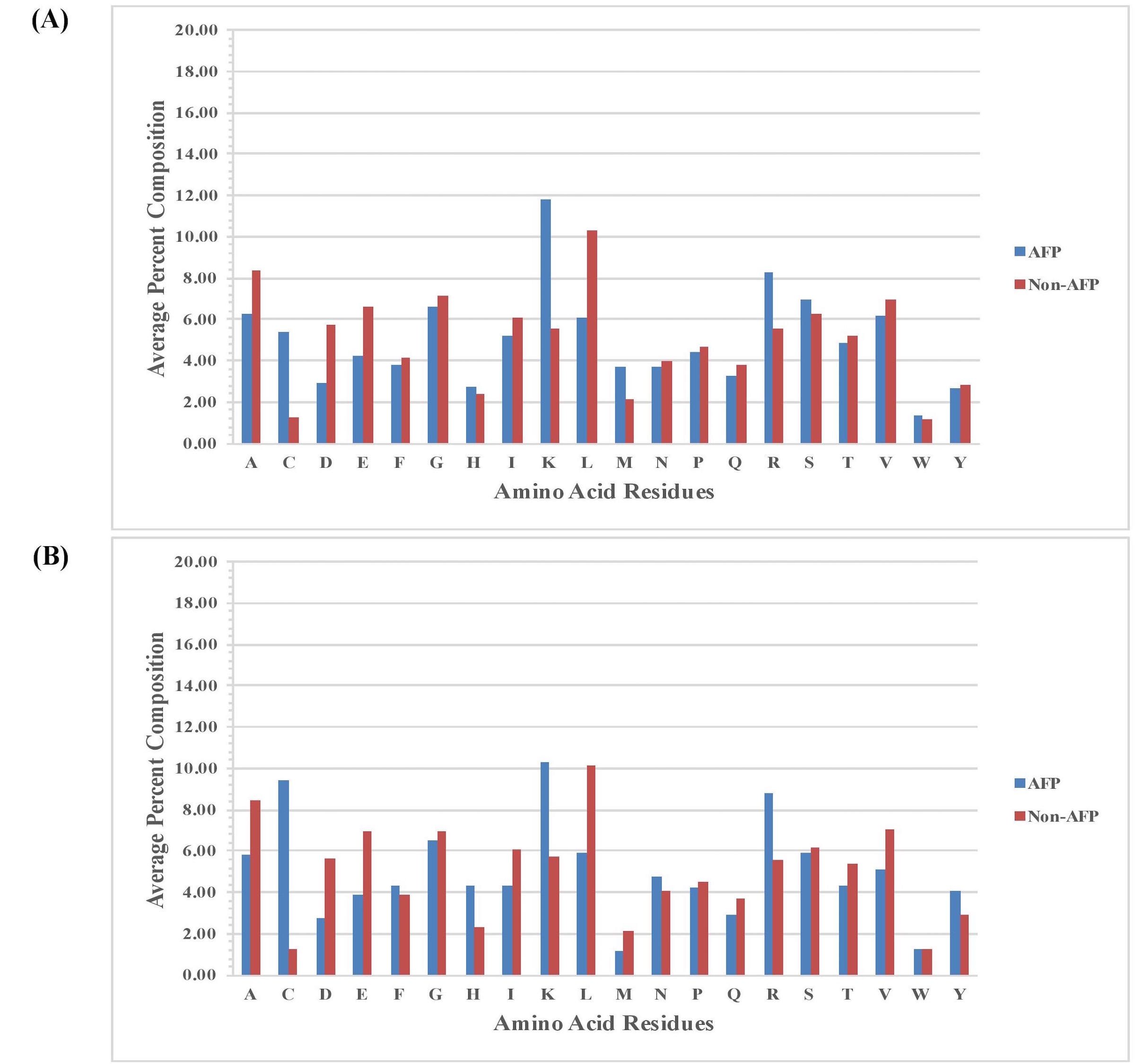
**

**Figure S4. Comparison of percent average amino acid composition of the (A) first 15 N-terminal and (B) first 15 C-terminal AFPs and non-AFPs of Antifp_Main.**

**
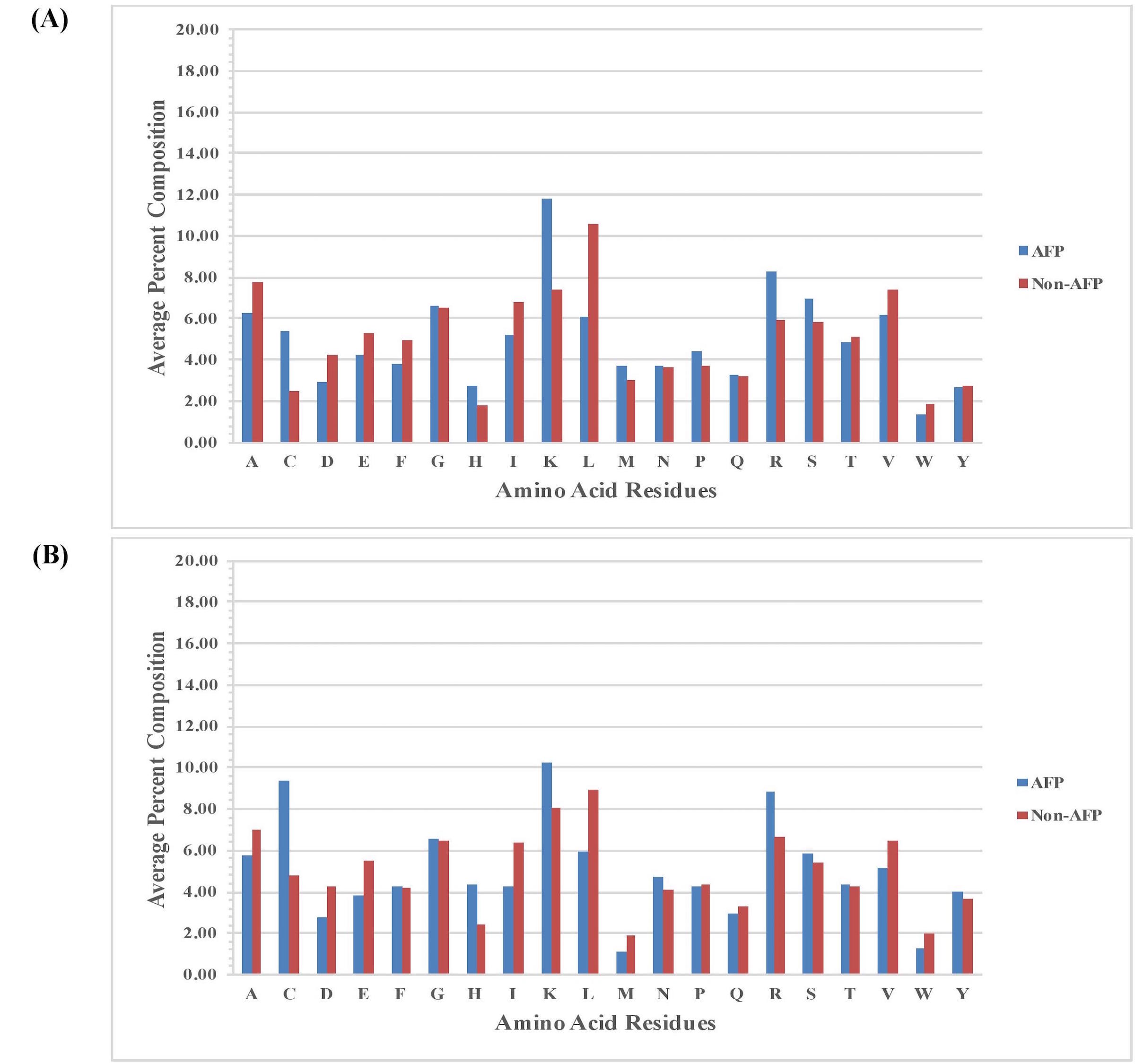
**

**Figure S5. Heat map illustrating the positional preference of each type of residue at (first 15 positions) N and C-terminus (A) positive and (B) negative data of Antifp_DS1.**


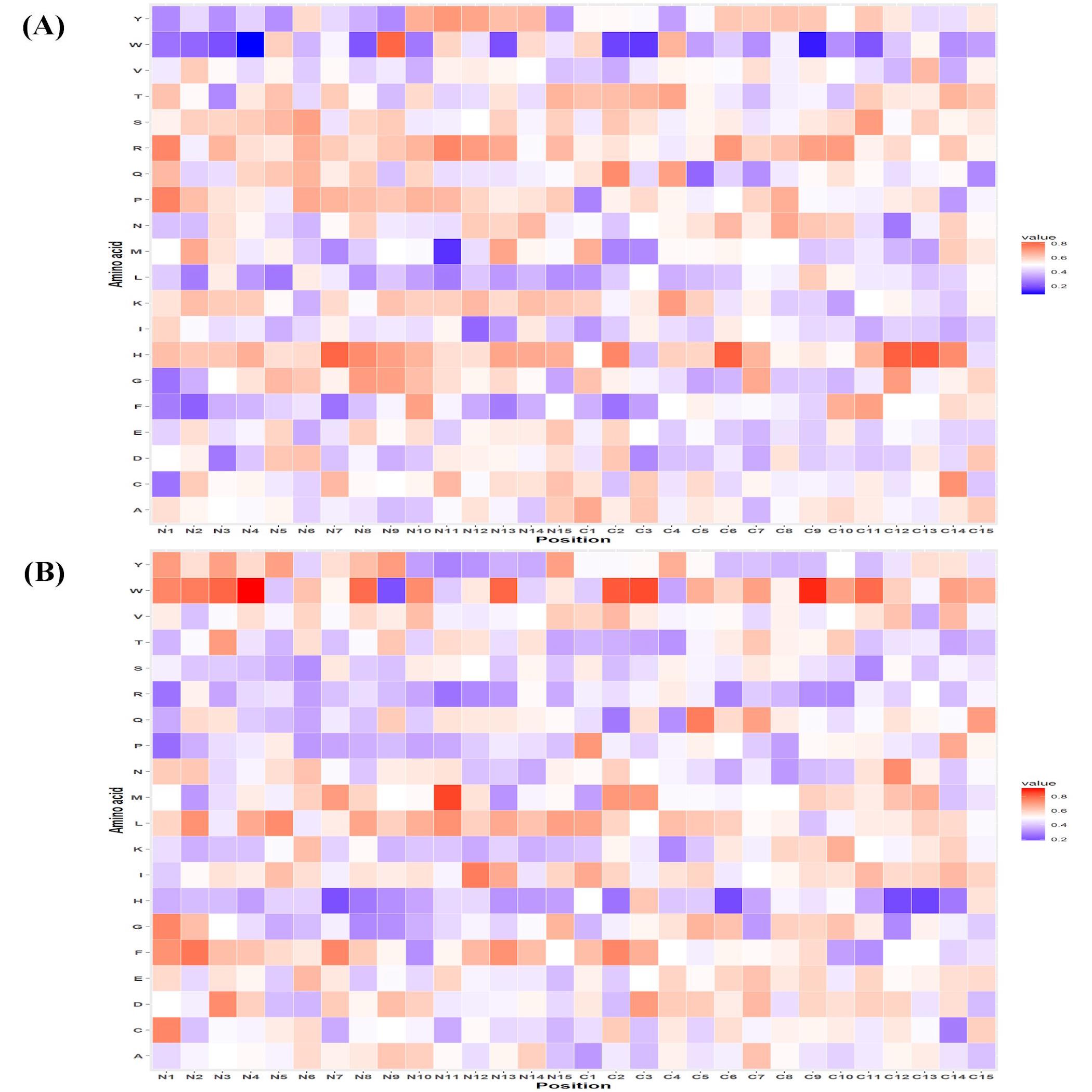


**Figure S6. Heat map illustrating the positional preference of each type of residue at (first 15 positions) N and C-terminus (A) positive and (B) negative data of Antifp_DS2.**

**
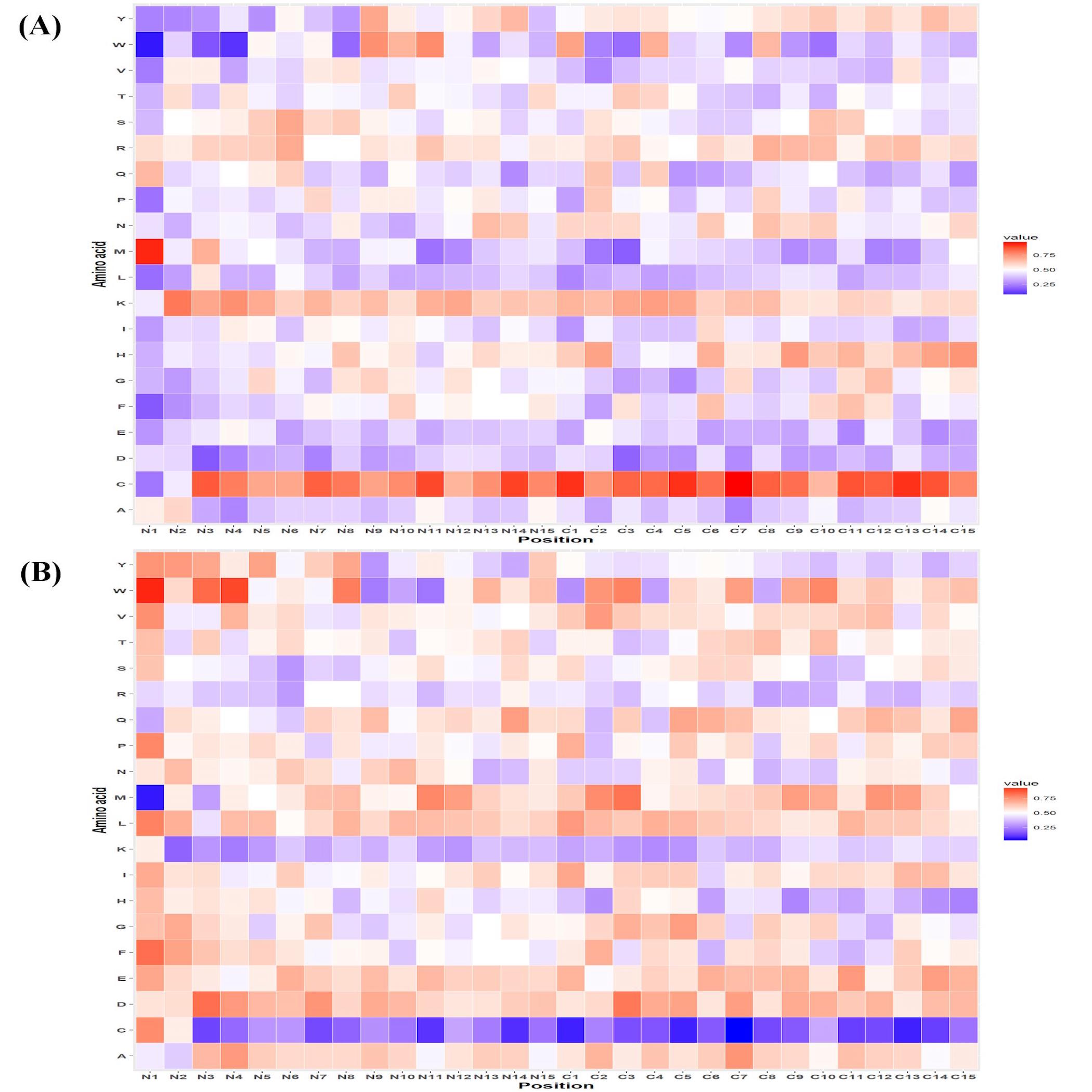
**
